# Mackler’s Triad: An Evolving Case of Boerhaave Syndrome in the Emergency Department

**DOI:** 10.7759/cureus.37978

**Published:** 2023-04-22

**Authors:** Izak A Loftus, Etimbuk E Umana, Izak P Scholtz, Deirdre McElwee

**Affiliations:** 1 Emergency Medicine, Mater Misericordiae University Hospital, Dublin, IRL

**Keywords:** mackler's triad, boerhaave syndrome, oesophageal tear, emergency medicine, geriatric medicine

## Abstract

An elderly lady, known with a background history of Alzheimer’s dementia, gastro-oesophageal reflux disease and a reported history of self-induced vomiting, presented to our emergency department with a two-day history of vomiting, diarrhoea, anorexia, and malaise. Initial clinical examination and investigations only demonstrated mild dehydration. Despite a satisfactory response to initial symptomatic treatment, with complete cessation of vomiting, the patient had a recent sudden deterioration. Due to continued forcible belching, it was found that she had developed a sudden onset of back pain and subcutaneous emphysema. A CT scan showed mid-oesophageal rupture along with pneumomediastinum and bilateral pneumothoraxes. The patient was subsequently diagnosed with Boerhaave syndrome. Due to her clinical factors and the risk of surgical management, it was decided that she should be managed non-operatively with oesophageal stenting and bilateral chest drains, which was met with a good clinical course and outcome.

## Introduction

Boerhaave syndrome, or rupture of the esophagus due to increased intraluminal oesophageal pressure, is mainly associated with vomiting and is most prevalent in middle-aged males with a strong history of alcohol consumption [1]. Patients may demonstrate vague symptoms or have chest pain, vomiting, and subcutaneous emphysema (the Mackler triad) [2]. Boerhaave syndrome is known for a high mortality rate of up to 40% [1,3]. Therefore swift and aggressive management is required to manage the perforation and to limit and treat soiling of the mediastinal and pleural cavities. A high index of suspicion is required to make a timely diagnosis and ensure rapid imaging to guide further interventions. However, due to the low incidence of this condition, there is still controversy regarding the ideal management [2,4,5].

## Case presentation

An elderly lady, known with Alzheimer's dementia, gastro-oesophageal reflux disease, hypertension, and a queried history of bulimia nervosa, presented to our emergency department with a two-day history of vomiting, diarrhea, anorexia, and malaise. Her pulse rate was 58 beats per minute, blood pressure 185/68 mmHg, temperature 37.2˚C, and oxygen saturation was 96% on room air. Although she reported a history of self-induced vomiting, her BMI was 26,7. During the initial clinical examination, she had mild dehydration and was actively vomiting watery food content. Apart from the above signs, she had no other remarkable findings on systemic examination.

Initial management consisted of intravenous antiemetics and fluids, met by complete cessation of active vomiting. A plain film chest x-ray, blood, and urinalysis were done as part of her initial workup. Throughout the subsequent time that passed, she had no further episodes of vomiting but continued to forcibly belch (a habit she has had for years, according to her next of kin).

Soon after returning from her chest x-ray, her relative notified us of a sudden onset of severe sharp right mid-back pain and voiced concern that her neck seemed enlarged. This prompted a repeat clinical examination, which demonstrated extensive subcutaneous emphysema along her neck and back and softening of her voice. Although she appeared to be in severe discomfort due to pain, she was calm and voiced that she did not have any shortness of breath.

Investigations

**Figure 1:**
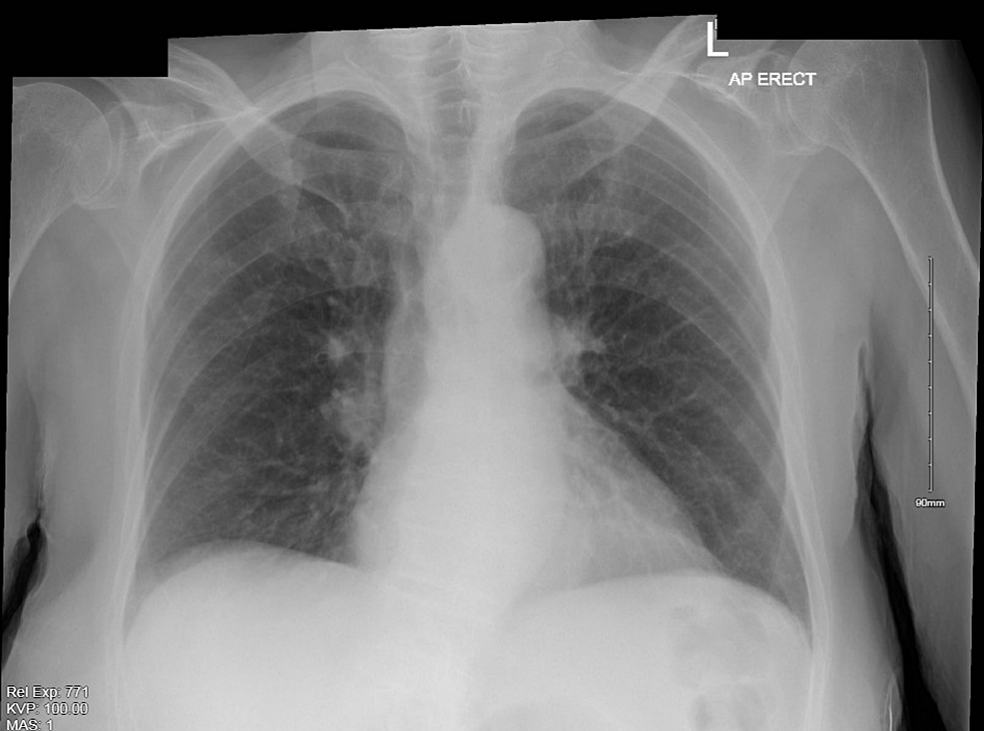
Plain Film Chest X-Ray with no Evidence of Pneumomediastinum or Pneumothorax

The initial chest x-ray, done about 45 minutes before the change in her clinical condition, showed no acute abnormalities, most notably no pneumothorax or pneumomediastinum (figure 1).

A hypochloraemic hypokalaemic metabolic alkalosis (pH 7.57; potassium 3.4 mmol/l; chloride 90 mmol/l, standard bicarbonate 40.8 mmol/l) was present on her initial blood gas. Her full blood count was normal and C-reactive protein was below 1 mg/L. Her renal profile demonstrated a similar electrolyte profile to her venous blood gas and mildly raised creatinine (89 umol/l), consistent with dehydration secondary to vomiting. A 12-lead ECG only showed a normal sinus rhythm.

After her change in condition and subsequent clinical examination, an emergent CT neck and chest was ordered to ascertain the cause of the subcutaneous emphysema.

**Figure 2:**
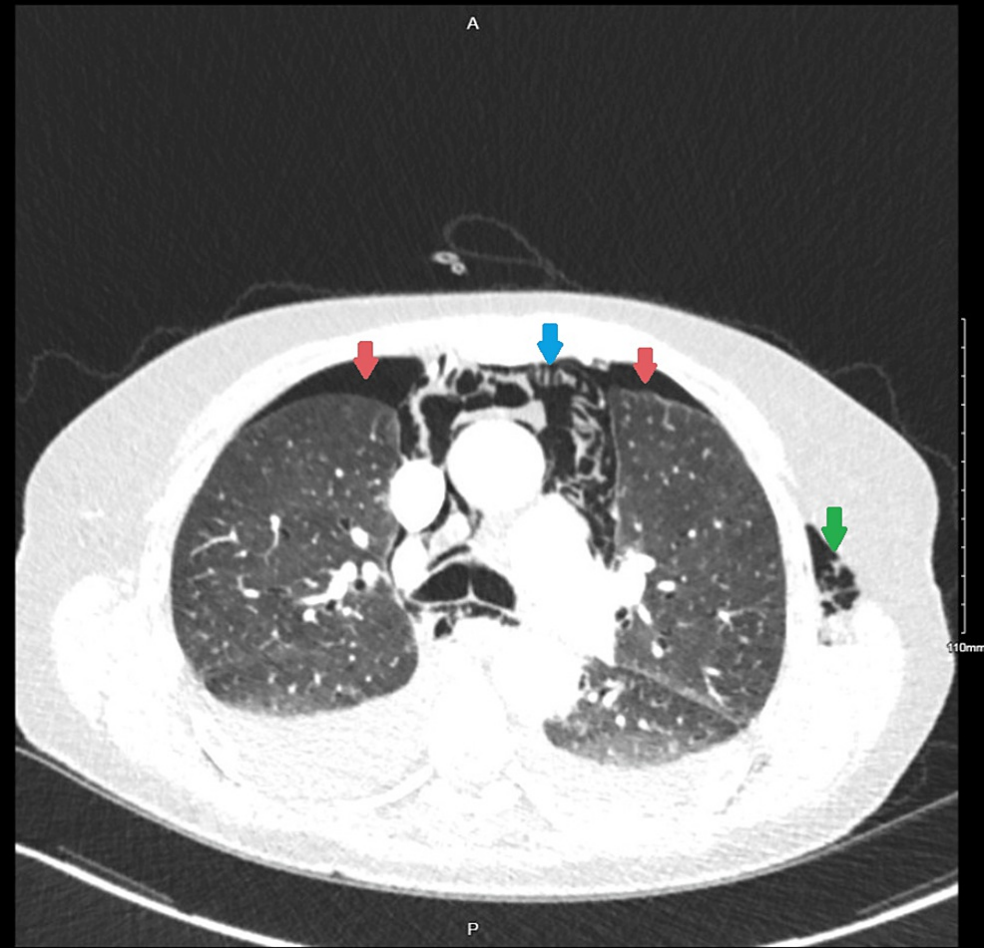
Transverse CT Thorax at the Level of the Carina with Evidence of Bilateral Pneumothoraces (Red Arrows), Pneumomediastinum (Blue Arrow), and Subcutaneous Emphysema on the Left (Green Arrow)

**Figure 3:**
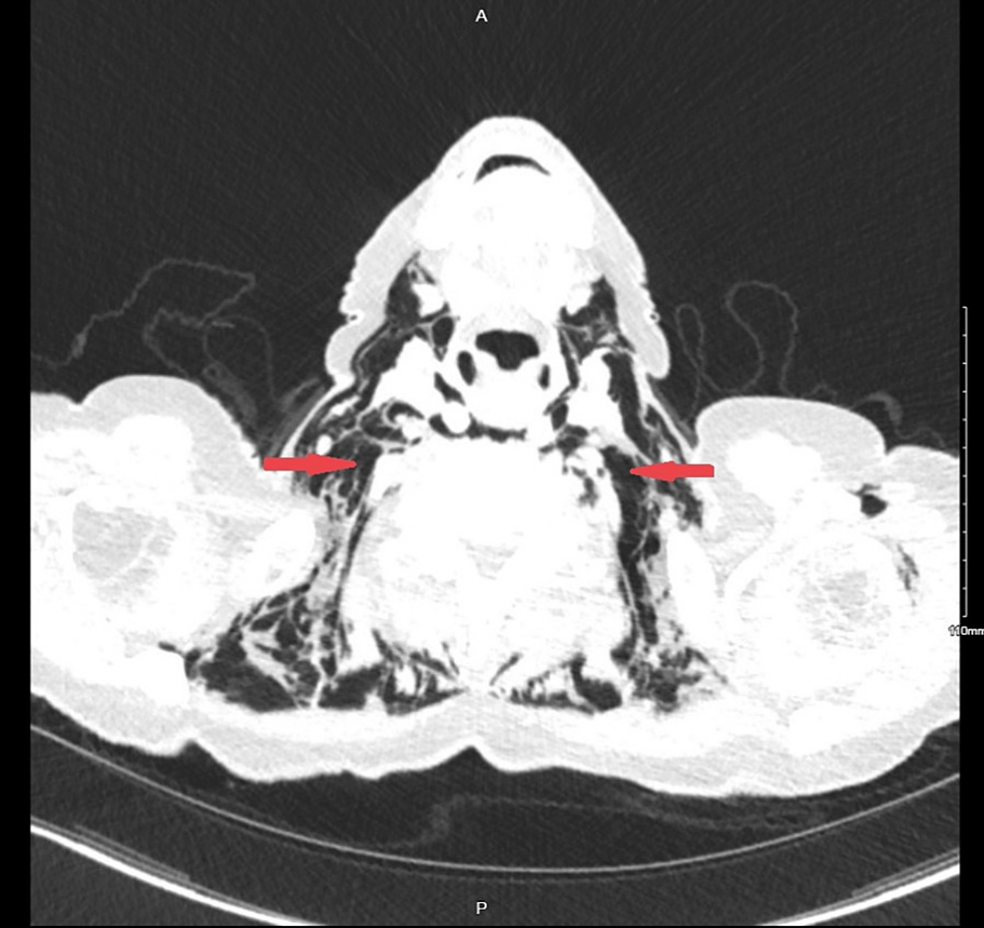
Transverse CT of the Neck with Extensive Subcutaneous Emphysema (Red Arrows Marking Dark Areas)

The neck and thorax CT assessment showed oesophageal rupture with the most likely site of rupture in the mid-esophagus (at the level of T8-9), as indicated by circumferential oesophageal thickening at this level. Furthermore, it demonstrated bilateral pneumothoraces, pneumopericardium, pneumomediastinum, and subcutaneous emphysema along the soft tissues of the chest, back, neck, and left arm (Figures 2-3).

Differential diagnosis

Vomiting and diarrhea are common presenting symptoms due to various causes. In this patient, the initial working diagnosis was guided by the identical presentation six weeks before she was admitted for urosepsis. Due to the short duration of symptoms and the limited findings on clinical examination, she was managed as a patient with mild dehydration secondary to acute gastroenteritis. At this stage, myocardial infarction or pancreatitis was still considered a potential underlying cause but was excluded based on a normal ECG and biochemical workup.

After the onset and progression of the new symptoms, the initial diagnosis and disease process was still managed with symptomatic treatment. However, the clinical team's full attention was shifted to identifying and treating the cause of the subcutaneous emphysema. Although spontaneous pneumothorax is more prevalent and was considered a potential cause due to the history of recent vomiting, back pain, and subcutaneous emphysema, the immediate clinical suspicion was an oesophageal rupture.

Treatment

After the CT scan, she was moved to a resuscitation bay for continued monitoring and care. At this stage, she was kept nil by mouth to avoid further soiling, and appropriate intravenous fluids and broad-spectrum antibiotics were commenced. Although comfortable and reported no respiratory distress, ICU/anesthetics were called to assess her airway instead of possible obstruction, as indicated by softening her voice. In addition, the general surgeons were consulted to review her regarding further definitive management. Bilateral size 16 French gauge Seldinger chest drains were inserted in the emergency department while awaiting ICU transfer.

Outcome and follow-up

She was transferred to our intensive care unit for continued monitoring and management. Shortly after arrival at ICU, she required intubation and ventilation due to an acute deterioration in her clinical condition. Upper endoscopy was performed on day two of admission while she was intubated, and an oesophageal stent was placed over a 15 mm perforation. She was extubated the following day and moved to the high-dependency unit for 20 days. 

Nutritional requirements were met with total parenteral nutrition during her first days of admission. The following nutritional requirements were then met by a combination of enteral (nasogastric) and parenteral nutrition, which poor compliance by the patient complicated, as she repeatedly removed her nasogastric tube. Before stent removal, she mainly was not taking anything by mouth but could tolerate a soft diet.

On day 50, a repeat upper endoscopy confirmed that the perforation site was healed, and the interval stent was removed. On day 56 a gastrograffin swallow was done, which showed no evidence of perforation, but identified gross dilatation of the esophagus at the site of the stent. After successfully tolerating a complete diet, she was discharged home on day 62 after admission. She was well and deemed fit for discharge at her follow-up appointment six weeks after discharge at the surgical outpatient department.

## Discussion

Boerhaave syndrome, or oesophageal rupture due to increased intraluminal oesophageal pressure, is a rare clinical syndrome with a high mortality of up to 40% [1,3], mostly attributed to severe local and systemic sepsis due to the leakage of food content from the esophagus. As in our case, a history of vomiting is the most frequent cause of spontaneous oesophageal perforation, but other events causing straining (such as childbirth, defecating, or weightlifting) can also lead to perforation [6]. This condition has a large predisposition towards men (82% of cases), and usually, patients are known with a history of significant alcohol intake (41% of cases) [7].

A literature review by Aiolfi et al. determined that most studies (95,8%) reported a tear in the left posterolateral distal esophagus [4]. However, in our case, the rupture site was at the mid-esophagus at the level of T8-9, as indicated by circumferential wall thickening. The propensity towards a tear in the distal esophagus is believed to be due to a decreased amount of supportive longitudinal muscle fibers and a weakened oesophageal wall secondary to many vessels and nerves entering it at that point [2].

Prompt diagnosis is essential, and further management is guided by the timing of presentation, the degree of sepsis, and the patient's clinical condition [8]. Although clinical suspicion is needed, imaging is essential to diagnose and guide further management definitively. The modalities available are plain film chest x-ray, CT, contrast oesophagram with gastrograffin, and direct visualization by endoscopy [2,3,9]. Recently, CT thorax has become the recommended modality to diagnose an oesophageal rupture and its complications [2,3,9]. In our case, the patient had a normal chest x-ray initially, as this was done just before the rupture. After the change in clinical condition, a CT thorax was done, with a resultant diagnosis of the rupture and its complications. The following morning endoscopy was done with the intent of inserting a stent, where the size of the defect could be determined more accurately. This procedure should be done cautiously in a suspected or confirmed oesophageal rupture to prevent iatrogenic rupture propagation [5].

Although controversy regarding the management still exists [2,4,5], De Schipper et al. performed a literature review and created a subsequent treatment algorithm [8]. As per their suggestion, patients with Boerhaave syndrome can be treated conservatively (intravenous antibiotics ± percutaneous drainage of abscesses), endoscopically (oesophageal stenting [5]), or operatively (by transthoracic or transhiatal approach [10]) [11]. Patients who present within 48 hours (early diagnosis) without features of sepsis should have endoscopic management first (with the possibility of progression to operative management if complications develop) [8]. Therefore, apart from managing the perforation, further management principles include control of mediastinal/pleural sepsis, removing gastric content, and managing nutrition (either by feeding the jejunostomy tube or total parenteral nutrition) [8]. Endoscopic stenting managed our patient's perforation, with subsequent removal after six weeks. As our patient required bilateral intercostal drains for pneumothoraces but never required the evacuation of gastric content from the pleural cavities, smaller-sized (16 French gauge) Seldinger chest drains were used to limit trauma.

During the Covid-19 pandemic, stringent measures had to be implemented to control the number of patients and family members in our departments to prevent overcrowding and subsequent viral spread. The recommendation is often that family members wait outside while their loved ones receive treatment. Our patient's favorable clinical outcome can be attributed to the early recognition of the oesophageal rupture after a change in condition was detected. Although she was being monitored in the emergency department, this change in condition was very mild in the initial stages and would have been missed had it not been for her relative. As her daughter was allowed to stay with the patient, who is known with cognitive impairment and could not express herself adequately, she could pick up an early, nondescript symptom: mild facial and neck swelling. Therefore, this emphasizes the importance of the family presence or getting collateral in any patient you feel cannot offer comprehensive and accurate information. In addition, it highlights the well-known fact that our patient's family members know them better than us and that we should take notice of all cues of concern.

## Conclusions

The Boerhaave syndrome presents with the Mackler triad (vomiting, thoracic pain, and subcutaneous emphysema) in only about 50% of cases; other causes of increased intra-oesophageal pressure, such as belching, must be considered. Furthermore, a high index of suspicion is essential to assist with early diagnosis, as a prompt diagnosis will decrease morbidity and mortality by limiting the degree of mediastinal and pleural soiling and resultant sepsis.

CT is the imaging modality of choice for the diagnosis of Boerhaave syndrome as well as its complications. Although many patients will require surgical interventions, conservative management in the setting of early diagnosis and no mediastinal soiling should be considered for patients with Boerhaave syndrome.
